# Supplementary material for: LYCEUM: learning to call copy number variants on low-coverage ancient genomes
Source: Bioinformatics. 2025 Jul 15;41(Suppl 1):i285–93. doi: 10.1093/bioinformatics/btaf244 (PMC12261418; doi:10.1093/bioinformatics/btaf244)
Supplement: btaf244_Supplementary_Data [file btaf244_supplementary_data.pdf]

Supplementary Material
for
LYCEUM: Learning to call copy number variants on low coverage ancient genomes

1 Supplementary Figures

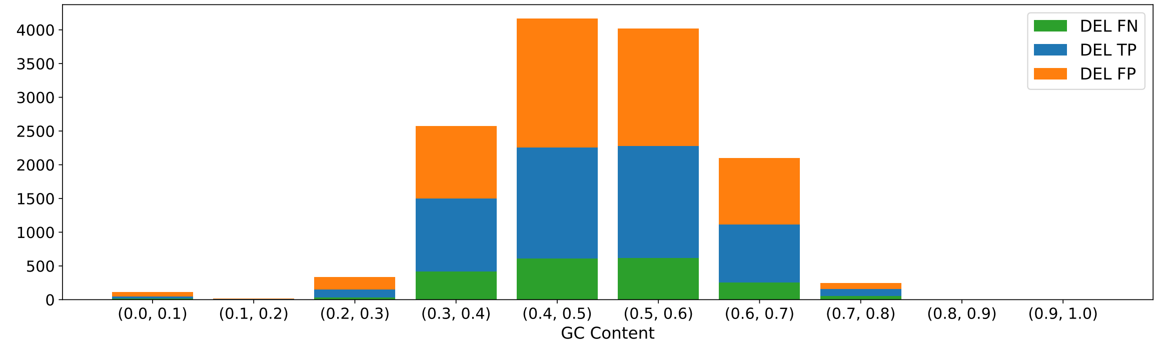

Supplementary Figure 1. Deletion performance of LYCEUM across varying GC content rates in real ancient samples at original coverage

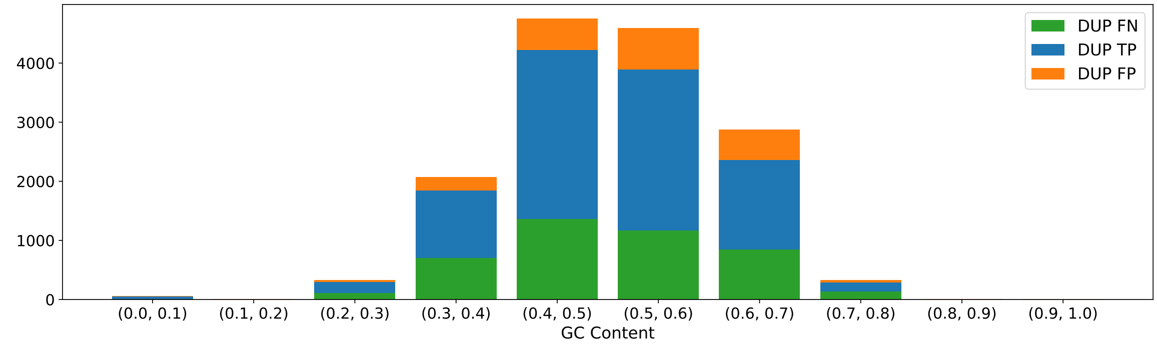

Supplementary Figure 2. Duplication performance of LYCEUM across varying GC content rates in real ancient samples at original coverage

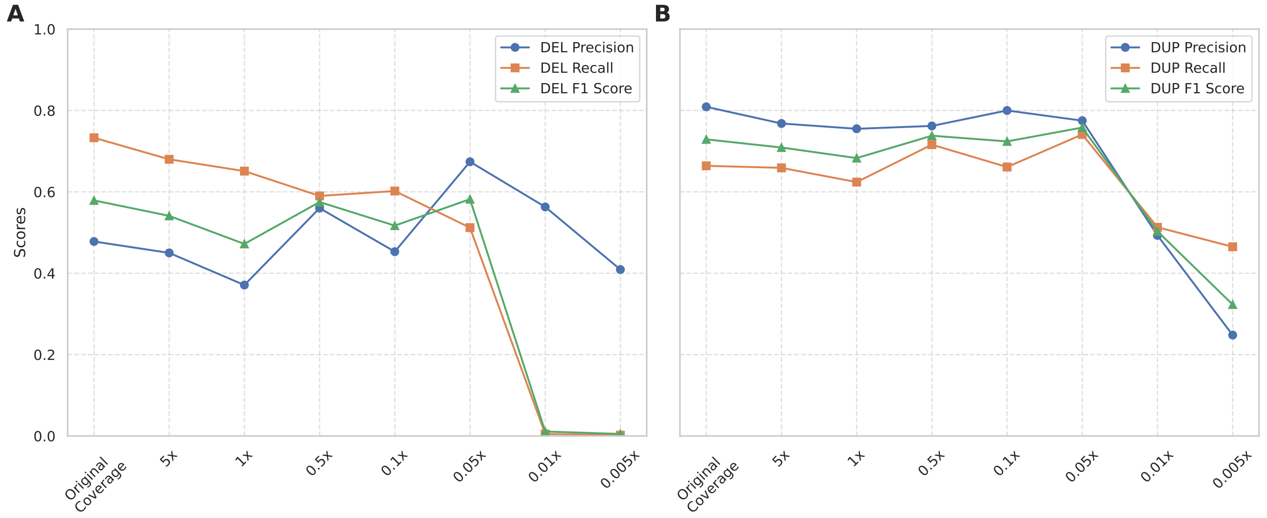

Supplementary Figure 3. Comparison of LYCEUM's performance in detecting deletion (A) and duplication (B) events in test samples across varying coverage levels, including low and ultra-low coverages in exon regions.

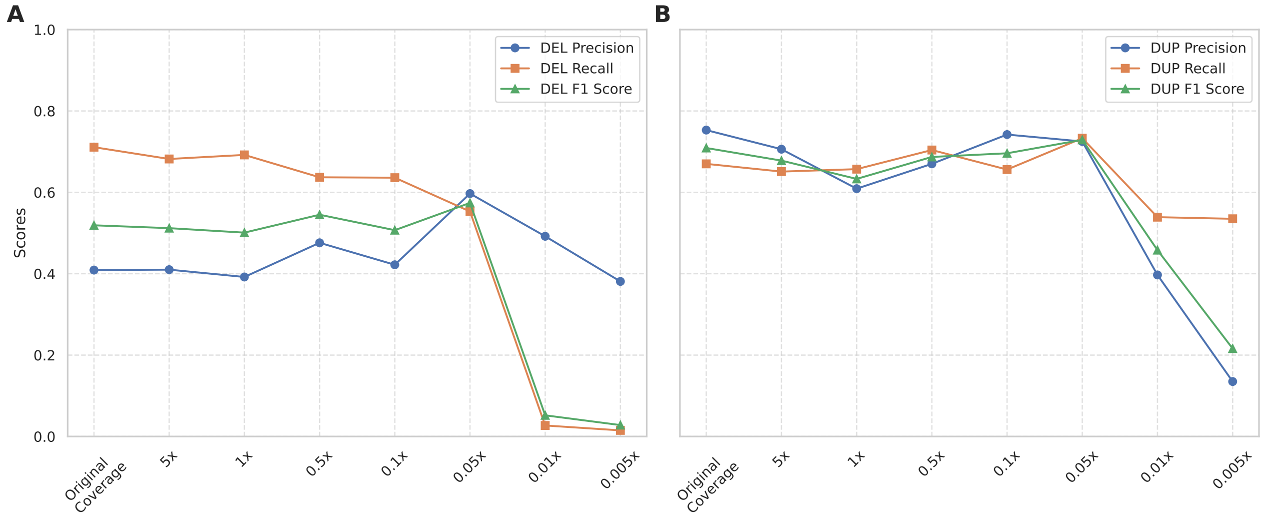

Supplementary Figure 4. Comparison of LYCEUM's performance in detecting deletion (A) and duplication (B) events in test samples across varying coverage levels, including low and ultra-low coverages in gene regions.

2 Supplementary Tables

Supplementary Table 1. List of 550 samples from the 1000 Genomes dataset selected for pre-training LYCEUM.

HG00472	HG01119	HG02190	HG01444	HG01113	HG00288	HG01860	HG00237	HG02023	HG02356
HG02292	HG01809	HG00622	HG02239	HG01918	HG01066	HG01372	HG01048	HG01173	HG01353
HG01468	HG00336	HG02089	HG01359	HG01606	HG01869	HG00879	HG00867	HG01111	HG01474
HG02079	HG01075	HG01857	HG02139	HG02230	HG01531	HG00473	HG02332	HG01051	HG01164
HG01605	HG01072	HG01198	HG01865	HG01253	HG01845	HG00736	HG01354	HG01802	HG01610
HG01187	HG01801	HG01049	HG01110	HG01485	HG00253	HG01598	HG01926	HG01088	HG01921
HG01398	HG02182	HG01360	HG02231	HG00421	HG00554	HG01497	HG01190	HG00242	HG00631
HG01847	HG01351	HG01783	HG01271	HG02262	HG01844	HG00331	HG00304	HG02307	HG00442
HG01914	HG01461	HG00096	HG01256	HG01507	HG01811	HG01489	HG02143	HG01509	HG00662
HG01069	HG01756	HG01492	HG02142	HG00252	HG00864	HG01956	HG00277	HG00419	HG01396
HG00332	HG01437	HG00367	HG01272	HG02238	HG00260	HG02156	HG00251	HG00614	HG02233
HG02075	HG01880	HG01958	HG01086	HG01063	HG01628	HG01840	HG01241	HG00590	HG00674
HG02069	HG00844	HG00268	HG01047	HG01571	HG01341	HG01205	HG00326	HG00632	HG01055
HG00250	HG02113	HG01870	HG01768	HG02072	HG02186	HG01085	HG01684	HG00099	HG01124
HG00583	HG01858	HG01572	HG01438	HG00422	HG01747	HG02090	HG02298	HG00325	HG02166
HG01623	HG00478	HG00436	HG00543	HG01257	HG01149	HG01920	HG01174	HG01441	HG00640
HG00278	HG02317	HG01710	HG02259	HG02061	HG00449	HG02084	HG02028	HG02006	HG01046
HG01200	HG01139	HG02009	HG01350	HG02085	HG01894	HG00464	HG01583	HG02284	HG02019
HG02291	HG02215	HG01125	HG01402	HG01848	HG01095	HG02282	HG01518	HG01079	HG02017
HG00334	HG00309	HG01953	HG01133	HG01284	HG01597	HG01501	HG00310	HG02322	HG02047
HG01630	HG01204	HG00382	HG00372	HG00653	HG01915	HG01326	HG00353	HG01512	HG01369
HG02285	HG00245	HG00551	HG00592	HG00634	HG00276	HG01248	HG00692	HG00280	HG02337
HG01893	HG00537	HG02081	HG01494	HG01777	HG02140	HG02051	HG01889	HG01440	HG00607
HG01790	HG01140	HG01805	HG01521	HG00536	HG01699	HG01176	HG01323	HG01362	HG01816
HG01851	HG01625	HG01631	HG00542	HG02070	HG00598	HG00595	HG00463	HG02050	HG00239
HG01412	HG02188	HG02064	HG02278	HG00256	HG02088	HG01191	HG00324	HG01992	HG01971
HG00663	HG00375	HG00327	HG01762	HG00534	HG01708	HG01890	HG01771	HG00560	HG02016
HG02082	HG01305	HG01668	HG00445	HG00384	HG02179	HG02185	HG01134	HG01935	HG02057
HG00448	HG01613	HG00361	HG01912	HG00345	HG00369	HG01148	HG01524	HG01593	HG01990
HG02151	HG01991	HG01058	HG01551	HG01694	HG01137	HG00357	HG01413	HG02147	HG01879
HG01883	HG00531	HG01131	HG01927	HG01988	HG00257	HG02223	HG02283	HG01866	HG02353
HG01941	HG01686	HG02232	HG00254	HG01171	HG01578	HG00623	HG00376	HG01259	HG01797
HG00321	HG01974	HG00732	HG00672	HG02138	HG01702	HG02054	HG00263	HG00360	HG01950
HG02277	HG01980	HG00329	HG01308	HG02343	HG01516	HG02309	HG01101	HG01620	HG00479
HG01073	HG01393	HG00404	HG00306	HG02219	HG02031	HG01603	HG02136	HG02104	HG01938
HG01789	HG01488	HG02058	HG00328	HG01456	HG01281	HG00701	HG00619	HG00475	HG00625
HG00271	HG00378	HG01348	HG00368	HG01673	HG00629	HG01432	HG01932	HG00656	HG02102
HG01600	HG01595	HG01112	HG00362	HG00476	HG00265	HG01612	HG00699	HG01682	HG01849
HG02299	HG01976	HG01817	HG00246	HG00236	HG01334	HG02165	HG00525	HG01356	HG01530
HG01061	HG01800	HG01810	HG01108	HG01345	HG00338	HG01363	HG00315	HG00234	HG00690
HG02272	HG01779	HG00410	HG02351	HG00596	HG00318	HG00452	HG01504	HG02060	HG01757
HG00743	HG01269	HG01250	HG01565	HG01589	HG01121	HG00500	HG01704	HG00599	HG02134
HG00232	HG02312	HG01525	HG02086	HG01162	HG01806	HG01871	HG02116	HG01550	HG01566
HG01855	HG00740	HG01965	HG00684	HG01945	HG00978	HG02152	HG01617	HG01031	HG01197
HG01670	HG01130	HG01375	HG01979	HG01462	HG01342	HG02153	HG00610	HG01776	HG00341
HG01970	HG01455	HG00255	HG02266	HG00379	HG01495	HG01815	HG01384	HG02256	HG00355
HG01459	HG00628	HG00956	HG01029	HG01182	HG02220	HG01798	HG01168	HG02330	HG00356
HG02286	HG00346	HG01812	HG02315	HG01503	HG01092	HG00313	HG02155	HG01924	HG00657
HG01968	HG01344	HG00350	HG01794	HG01161	HG00766	HG00611	HG02073	HG01951	HG02164
HG01142	HG00409	HG00403	HG00698	HG02053	HG00238	HG00581	HG01107	HG02187	HG01765
HG01859	HG01882	HG01868	HG00351	HG01506	HG02128	HG01537	HG02040	HG01357	HG00281
HG02250	HG01302	HG00428	HG00593	HG00290	HG02137	HG02325	HG01105	HG01077	HG00734
HG01491	HG02314	HG02002	HG00566	HG01247	HG01709	HG02095	HG02048	HG00689	HG02265
HG00330	HG00358	HG02076	HG01447	HG00097	HG01556	HG00261	HG01136	HG00373	HG01365
HG01094	HG01863	HG01104	HG01700	HG01936	HG02345	HG01366	HG00443	HG00337	HG01389

Supplementary Table 2. Metadata for 13 moderate-coverage ancient samples used in the fine-tuning of LYCEUM. [1–7].

Sample_ID	ID_publication	Population Name	Country	Data Type	Publication	Genome Coverage	Date	Latitude	Longitude
**Anzick-1**	Anzick-1	Anzick-1	USA	Shotgun	RasmussenNature2014	11,915	10776-10622 calBCE	45,993056	-110,66139
**BAR25**	BAR25	Anatolia_N	Turkey	Shotgun	MarchibioRxiv2020	12,65	8384-8205 calBP	40,3	29,56666
**BOT2016**	BOT2016	Botai	Kazakhstan	Shotgun	DamgaardScience2018	12,991	3632-3368 calBCE (4695 ±50 BP, UBA-32666)	53,305983	67,648166
**irk034**	irk034	Siberia_Cis_Baikal_LN	Russia	Shotgun	KılınçSciAdv2021	14,5	3645-3521 calBCE	53,2206944	103,39475
**Kotias**	Kotias	CHG	Georgia	Shotgun	JonesNatureCommunications2015	11,5591	7940-7600 calBCE [7938-7580 calBCE (8665 ±65 BP, RTT-5246), 7946-7612 calBCE (8745 ±40, OxA-28256)]	42,28	43,28
**LBK**	LBK	Stuttgart.SG	Germany	Shotgun	LazaridisNature2014	14,267	5310-5070 calBCE (6246 ±30 BP, MAMS-24635)	48,78	9,18
**LEPE48**	LEPE48	Iron_Gates_HG_EN	Serbia	Shotgun	MarchibioRxiv2020	10,92	8012-7867 calBP	44,552924	22,027563
**LEPE52**	LEPE52	Iron Gates E-MN	Serbia	Shotgun	MarchibioRxiv2020	12,37	7931-7693 calBP	44,552924	22,027563
**Loschbour**	Loschbour_published.DG	Loschbour.SG	Luxembourg	Shotgun	LazaridisNature2014	15,526	6210-5990 calBCE (7205 ±50 BP, OxA-7738)	49,81	6,4
**Nea2**	Nea2	Greece_N	Greece	Shotgun	MarchibioRxiv2020	12,51	8173-8023 calBP	40,5879	22,2512
**VC3-2**	VC3-2	Starcevo_EN	Serbia	Shotgun	MarchibioRxiv2020	11,22	7565-7426 calBP	44,762	20,6232
**WC1**	WC1	Iran_WezmehCave_N	Iran	Shotgun	BroushakiScience2016	9,15617	7455-7082 calBCE (8240 ±56 BP, UBA-25840)	34,6129	47,1057
**Yamnaya**	Yamnaya	Russia_EBA	Russia	Shotgun	DamgaardScience2018	23,312	3018-2887 calBCE (4315 ±34 BP, UBA-32667)	49,134217	75,851817

Supplementary Table 3. Metadata for 7 moderate coverage ancient samples used in 'Comparative performance analysis on simulated ancient genomic data' section to evaluate and compare the performance of LYCEUM with other methods [2, 5, 8].

Sample_ID	ID_publication	Population Name	Country	Data Type	Publication	Genome Coverage	Date	Latitude	Longitude
**AKT16**	AKT16	Anatolia_EN	Turkey	Shotgun	MarchibioRxiv2020	12,25	8635-8460 calBP	40,16707	28,7756103
**Bichon**	Bichon.SG	Bichon	Switzerland	Shotgun	JonesNatureCommunications2015	13,521	11820-11610 calBCE (11855 ±50 BP, OxA-27763)	47,0999985	6,86999989
**Nea3**	Nea3	Greece_N	Greece	Shotgun	MarchibioRxiv2020	11,57	8327-8040 calBP	40,5879	22,2512
**STAR1**	STAR1	Starcevo_EN	Serbia	Shotgun	MarchibioRxiv2020	10,55	7589-7476 calBP	44,8218	20,7299
**Ust**	Ust_Ishim_published.DG	Ust_Ishim	Russia	Shotgun	FuNature2014	26,345	45530-40610 calBCE [46064-40920 calBCE (41400 ±1300 BP, OxA-25516), 46364-40844 calBCE (41400 ±1400 BP, OxA-30190)]	57,7	71,1
**VLASA32**	VLASA32	Iron_Gates_HG	Serbia	Shotgun	MarchibioRxiv2020	12,65	9741-9468 calBP	44,53	22,05
**VLASA7**	VLASA7	Iron Gates LM	VLASA7	Shotgun	MarchibioRxiv2020	15,21	8764-8340 calBP	44,53	22,05

Supplementary Table 4. Metadata for 50 ancient samples used in analyses described in 'Segmental deletions detected by LYCEUM recover the demographic history' and 'Segmental deletions are negatively selected' sections. [1–19]

Sample_ID	ID_publication	Population Name	Country	Data Type	Publication	Genome Coverage	Date	Latitude	Longitude
**AKT16**	AKT16	Anatolia_EN	Turkey	Shotgun	MarchibioRxiv2020	12,25	8635-8460 calBP	40,16707	28,77561
**Ash128**	Ash128	Anatolia_EN	Turkey	Shotgun	YakaCurrentBiology2021	5,03217	8240-7941 (%95,4)	38,34	34,23
**ATP2**	ATP2.SG	Iberia_Chl	Spain	Shotgun	GuntherPNAS2015	4,080	2900-2679 calBCE (4210 ±30 BP, Beta-386394)	42,3525	-3,51833
**Bar31**	Bar31.SG	Anatolian_N	Turkey	Shotgun	HofmanovaPNAS2016	3,374	6419-6238 calBCE (7457 ±44 BP, UBA-29838)	40,3	29,56667
**Bar8**	Bar8.SG	Anatolian_N	Turkey	Shotgun	HofmanovaPNAS2016	6,285	6212-6030 calBCE (7238 ±38 BP, UBA-29837)	40,3	29,56667
**Bichon**	Bichon.SG	Bichon	Switzerland	Shotgun	JonesNatureCommunications2015	13,521	11820-11610 calBCE (11855 ±50 BP, OxA-27763)	47,1	6,87
**Bon002**	Bon002.SG	Boncuklu	Turkey	Whole Genome Capture	KilincCurrentBiology2016	6,688	8279-7977 calBCE (Wk-29763)	37,75191	32,8649
**BOT14**	BOT14	Botai	Kazakhstan	Shotgun	DamgaardScience2018	3,570	3518-3109 calBCE (4598 ±46 BP, UBA-32662)	53,30598	67,64817
**BOT15**	BOT15	Botai	Kazakhstan	Shotgun	DamgaardScience2018	2,895	3343-3026 calBCE (4474 ±37 BP, UBA-32663)	53,30598	67,64817
**Chan**	Chan.SG	Iberia_HG	Spain	Shotgun	GonzalesFortesCurrentBiology2017	1,580	7305-7057 calBCE (8155 ±42 BP, Ua-13398/Ua-38115)	43,14516	-7,04093
**CovaMoura9B**	CM9B	Portugal_LN_Chl	Portugal	Shotgun	MartinianoPLoSGenetics2017	2,25947	3700-2200 BCE	38,74504	-9,21522
**DA100**	DA100	TianShanHun	Kyrgyzstan	Shotgun	DamgaardNature2018	1,902	178-423 calCE (1719 ±48 BP, UBA-31213)	42,15667	77,40556
**DA222**	DA222	Karluk	Kyrgyzstan	Shotgun	DamgaardNature2018	3,269	750-950 CE	43,2025	76,98167
**DA243**	DA243	Alan	Russia	Shotgun	DamgaardNature2018	2,780	450-1350 CE	43,95865	42,5873
**DA249**	DA249	Shamanka_EN	Russia	Shotgun	DamgaardScience2018	4,374	5988-5791 calBCE (7005 ±40 BP, OxA-21535)	51,69833	103,7031
**DA28**	DA28	GoldenHordeAsian	Kazakhstan	Shotgun	DamgaardNature2018	3,682	1200-1600 CE	46,99722	66,28583
**DA379**	DA379	Namazga_CA	Turkmenistan	Shotgun	DamgaardScience2018	0,0434962	3370-3023 calBCE (4515 ±57 BP, UBA-33658)	37,60098	59,32841
**DA380**	DA380	Namazga_CA	Turkmenistan	Shotgun	DamgaardScience2018	0,444332	3364-3097 calBCE (4528 ±40 BP, UBA-33659)	37,60098	59,32841
**DA45**	DA45	XiongNu	Mongolia	Shotgun	DamgaardNature2018	7,960	183-41 calBCE (2083 ±27 BP, UBA-31155)	42,52583	105,18
**EBA1**	EBA1	Central_Steppe_EMBA	Russia	Shotgun	DamgaardScience2018	3,885	2286-2037 calBCE (3752 ±35 BP, UBA-32665)	51,63134	74,66445
**EBA2**	EBA2	Central_Steppe_EMBA	Russia	Shotgun	DamgaardScience2018	8,814	2620-2468 calBCE (4013 ±33 BP, UBA-32664)	52,62978	76,72765
**GB**	GB.SG	Iron_Gates_EN	Romania	Shotgun	GonzalesFortesCurrentBiology2017	3,370	3506-3349 calBCE (4621 ±28 BP, MAMS-28614)	46,52616	26,92981
**Klei10**	Klei10	Greece_Klei10	Greece	Shotgun	HofmanovaPNAS2016	1,91646	4230-3995 calBCE (5559 ±22 BP, MAMS-23038)	40,43	21,78
**LugarCanto42**	LC42	Portugal_MN	Portugal	Shotgun	MartinianoPLoSGenetics2017	2,60478	4500-3500 BCE	39,00812	9,390754
**LugarCanto44**	LC44	Portugal_MN	Portugal	Shotgun	MartinianoPLoSGenetics2017	1,77033	4500-3500 BCE	39,00812	9,390754
**Motala12**	Motala12	Motala12_SG	Sweden	Shotgun	LazaridisNature2014	1,92948	5722-5628 calBCE (6773 ±30 BP, Ua-51723)	58,535	15,046
**Nea3**	Nea3	Greece_N	Greece	Shotgun	MarchibioRxiv2020	11,57	8327-8040 calBP	40,5879	22,2512
**NEO240**	NEO240	Neolithic	Russia	Shotgun	SikoraNature2019	6,16589	5625-5481 calBCE (6627 ±45 BP, UBA-33768)	44,5	135,4
**R1**	R1	Italian_LBA-EIA	Italy	Shotgun	AntonioScience2019	3,669	930 - 839 calBCE	42,88014	13,89377
**R2**	R2	Italian_EN	Italy	Shotgun	AntonioScience2019	3,330	6066 - 6002 calBCE	41,96	13,54
**R3**	R3	Italian_UPP	Italy	Shotgun	AntonioScience2019	3,702	5836 - 5723 calBCE	41,96	13,54
**R4**	R4	Italian_UPN	Italy	Shotgun	AntonioScience2019	3,309	2950 - 2880 calBCE	41,96	13,54
**R7**	R7	Italian_UPN	Italy	Shotgun	AntonioScience2019	2,673	8821 - 8642 calBCE	41,96	13,54
**R9**	R9	Italian_EN	Italy	Shotgun	AntonioScience2019	3,453	5607 - 5485 calBCE	41,96	13,54
**RISE493**	RISE493	Karasut	Russia	Shotgun	AllentoftNature2015	4,972	1531-1427 calBCE (3214 ±26 BP, OxA-31211)	53,152	91,05
**RISE495**	RISE495	Karasut	Russia	Shotgun	AllentoftNature2015	2,996	1400-900 BCE	52,954	90,187
**RISE496**	RISE496	Karasut	Russia	Shotgun	AllentoftNature2015	1,599	1414-1261 calBCE (3070 ±28 BP, OxA-31213)	52,954	90,187
**RISE497**	RISE497	Karasut	Russia	Shotgun	AllentoftNature2015	5,507	1400-900 BCE	52,954	90,187
**RISE505**	RISE505	Adronovo	Russia	Shotgun	AllentoftNature2015	3,482	1746-1626 calBCE (3391 ±27 BP, OxA-31216)	53,456	85,447
**RISE511**	RISE511	Afanasievo	Russia	Shotgun	AllentoftNature2015	2,448	2909-2679 calBCE (4224 ±36 BP, OxA-31568)	54,584	90,775
**RISE664**	RISE664	Okunevo_EMBA	Russia	Shotgun	DamgaardScience2018	4,261	2459-2206 calBCE (3850 ±38 BP, UBA-31593)	53,54781	91,02565
**RISE675**	RISE675	Okunevo_EMBA	Russia	Shotgun	DamgaardScience2018	0,503469	2859-2350 calBCE (4023 ±56 BP, UBA-31597)	53,70856	90,35981
**RISE98**	RISE98	Nordic_LN	Sweden	Shotgun	AllentoftNature2015	4,670	2275-2032 calBCE (3736 ±32 BP, OxA-28987)	55,381	13,445
**SC2**	SC2	Iron_Gates_HG.SG	Serbia-romania	Shotgun	GonzalesFortesCurrentBiology2017	2,69884	7250-6500 BCE	44,62971	22,61256
**scy009**	scy009	Scythian	Ukraine	Shotgun	KrzewińskaScienceAdvances2018	2,11486	768-431 calBCE (2470 ±30 BP, Beta-451570)	46,33283	29,46166
**STAR1**	STAR1	Starcevo_EN	Serbia	Shotgun	MarchibioRxiv2020	10,55	7589-7476 calBP	44,8218	20,7299
**Ust**	Ust_Ishim_published.DG	Ust_Ishim	Russia	Shotgun	FuNature2014	26,345	45530-40610 calBCE [46064-40920 calBCE (41400 ±1300 BP, OxA-25516), 46364-40844 calBCE (41400 ±1400 BP, OxA-30190)]	57,7	71,1
**VLASA32**	VLASA32	Iron_Gates_HG	Serbia	Shotgun	MarchibioRxiv2020	12,65	9741-9468 calBP	44,53	22,05
**VLASA7**	VLASA7	Iron Gates LM	VLASA7	Shotgun	MarchibioRxiv2020	15,21	8764-8340 calBP	44,53	22,05
**Yana**	Yana1	Upper-Paleolithic	Russia	Shotgun	SikoraNature2019	20,4714	30250-29550 BCE	70,7	135,4

Supplementary Table 5. Runtime comparison of LYCEUM versus other evaluated CNV callers on 5× coverage.

Tool	Runtime (h:m / genome)
**CNVnator**	0:21
**CONGA**	0:10
**CONTROL-FREEC**	0:45
**GATK**	0:55
**LYCEUM**	**0:05**

Supplementary Table 6. The performance comparison of the CNV callers on the down-sampled ancient samples (test set) in exon regions. CNVnator calls on original coverage samples are used as the ground truth.

Coverage	Tools	DEL Precision	DEL Recall	DEL F1 Score	DUP Precision	DUP Recall	DUP F1 Score	Overall Precision	Overall Recall	Overall F1 Score
Original Coverage	CNVnator	1.0	1.0	1.0	1.0	1.0	1.0	1.0	1.0	1.0
	CONGA	0.838	0.093	0.167	0.528	0.009	0.017	0.683	0.051	0.092
	Control-FREEC	0.176	0.826	0.291	0.861	0.814	0.837	0.5185	0.82	0.564
	GATK	0.133	0.89	0.231	0.233	0.013	0.025	0.183	0.4515	0.128
	LYCEUM	0.478	0.733	0.579	0.809	0.664	0.729	0.644	0.699	0.654
5x	CNVnator	0.928	0.913	0.92	0.914	0.826	0.868	0.921	0.87	0.894
	CONGA	0.835	0.094	0.169	0.533	0.008	0.016	0.684	0.051	0.0925
	Control-FREEC	0.176	0.829	0.29	0.889	0.804	0.845	0.5325	0.8165	0.5675
	GATK	0.15	0.588	0.239	0.091	0.005	0.009	0.1205	0.2965	0.124
	LYCEUM	0.45	0.68	0.541	0.768	0.659	0.709	0.609	0.67	0.625
1x	CNVnator	0.843	0.779	0.81	0.879	0.464	0.607	0.861	0.622	0.709
	CONGA	0.769	0.097	0.173	0.504	0.009	0.017	0.6365	0.053	0.095
	Control-FREEC	0.163	0.765	0.268	0.845	0.694	0.762	0.504	0.7295	0.515
	GATK	0.145	0.554	0.23	0.009	0.002	0.004	0.077	0.278	0.117
	LYCEUM	0.371	0.651	0.472	0.755	0.624	0.683	0.563	0.638	0.578
0.5x	CNVnator	0.785	0.671	0.723	0.76	0.252	0.378	0.773	0.462	0.551
	CONGA	0.639	0.097	0.168	0.479	0.008	0.016	0.559	0.0525	0.092
	Control-FREEC	0.143	0.655	0.235	0.828	0.593	0.691	0.4855	0.624	0.463
	GATK	0.128	0.535	0.207	0.002	0.004	0.002	0.065	0.2695	0.105
	LYCEUM	0.56	0.59	0.575	0.762	0.716	0.738	0.661	0.653	0.657
0.1x	CNVnator	0.66	0.418	0.512	0.363	0.037	0.067	0.512	0.228	0.29
	CONGA	0.266	0.096	0.141	0.089	0.006	0.011	0.1775	0.051	0.076
	Control-FREEC	0.101	0.44	0.164	0.626	0.347	0.446	0.3635	0.3935	0.305
	GATK	0.069	0.423	0.119	0.0	0.001	0.0	0.0345	0.212	0.06
	LYCEUM	0.453	0.602	0.517	0.8	0.661	0.724	0.627	0.632	0.621
0.05x	CNVnator	0.709	0.362	0.479	0.99	0.008	0.015	0.85	0.185	0.247
	CONGA	0.161	0.086	0.112	0.038	0.004	0.008	0.0995	0.045	0.06
	Control-FREEC	0.092	0.39	0.148	0.518	0.308	0.386	0.305	0.349	0.267
	GATK	0.052	0.408	0.093	0.0	0.001	0.0	0.026	0.2045	0.047
	LYCEUM	0.674	0.512	0.582	0.775	0.741	0.758	0.725	0.627	0.67

Supplementary Table 7. The performance comparison of the CNV callers on the down-sampled ancient samples (test set) in gene regions. CNVnator calls on original coverage samples are used as the ground truth.

Coverage	Tools	DEL Precision	DEL Recall	DEL F1 Score	DUP Precision	DUP Recall	DUP F1 Score	Overall Precision	Overall Recall	Overall F1 Score
Original Coverage	CNVnator	1.0	1.0	1.0	1.0	1.0	1.0	1.0	1.0	1.0
	CONGA	0.747	0.124	0.213	0.559	0.015	0.028	0.653	0.0695	0.1205
	Control-FREEC	0.2	0.741	0.315	0.867	0.786	0.824	0.534	0.764	0.57
	GATK	0.192	0.415	0.263	0.143	0.003	0.006	0.1675	0.209	0.1345
	LYCEUM	0.409	0.711	0.519	0.753	0.67	0.709	0.581	0.691	0.614
5x	CNVnator	0.905	0.853	0.878	0.917	0.802	0.856	0.911	0.828	0.867
	CONGA	0.742	0.124	0.212	0.562	0.014	0.027	0.652	0.069	0.1195
	Control-FREEC	0.199	0.742	0.314	0.887	0.762	0.82	0.543	0.752	0.567
	GATK	0.272	0.339	0.302	0.054	0.002	0.004	0.163	0.1705	0.153
	LYCEUM	0.41	0.682	0.512	0.706	0.651	0.678	0.558	0.667	0.595
1x	CNVnator	0.82	0.676	0.741	0.883	0.369	0.52	0.852	0.523	0.631
	CONGA	0.592	0.126	0.208	0.528	0.015	0.028	0.56	0.0705	0.118
	Control-FREEC	0.174	0.636	0.273	0.852	0.638	0.73	0.513	0.637	0.502
	GATK	0.267	0.317	0.29	0.0	0.0	nan	0.1335	0.1585	0.29
	LYCEUM	0.392	0.692	0.501	0.609	0.657	0.633	0.501	0.675	0.567
0.5x	CNVnator	0.713	0.543	0.616	0.713	0.219	0.335	0.713	0.381	0.476
	CONGA	0.416	0.123	0.189	0.45	0.014	0.027	0.433	0.0685	0.108
	Control-FREEC	0.149	0.527	0.233	0.824	0.491	0.616	0.487	0.509	0.425
	GATK	0.231	0.314	0.266	0.004	0.004	0.004	0.1175	0.159	0.135
	LYCEUM	0.476	0.637	0.545	0.67	0.704	0.687	0.573	0.671	0.616
0.1x	CNVnator	0.649	0.297	0.408	0.41	0.051	0.09	0.53	0.174	0.249
	CONGA	0.133	0.126	0.129	0.071	0.011	0.02	0.102	0.0685	0.0745
	Control-FREEC	0.099	0.335	0.153	0.55	0.276	0.367	0.325	0.306	0.26
	GATK	0.116	0.232	0.155	0.0	0.001	0.001	0.058	0.1165	0.078
	LYCEUM	0.422	0.636	0.507	0.742	0.656	0.696	0.582	0.646	0.602
0.05x	CNVnator	0.68	0.259	0.375	1.0	0.013	0.026	0.84	0.136	0.201
	CONGA	0.078	0.109	0.091	0.03	0.008	0.012	0.054	0.0585	0.0515
	Control-FREEC	0.085	0.278	0.13	0.454	0.266	0.336	0.27	0.272	0.233
	GATK	0.081	0.231	0.12	0.001	0.001	0.001	0.041	0.116	0.0605
	LYCEUM	0.597	0.553	0.574	0.725	0.733	0.729	0.661	0.643	0.652

Supplementary Table 8. Confusion Matrices for the performance comparison of CNVnator on down-sampled ancient samples (test set) in exon regions. CNVnator calls on original coverage samples are used as the ground truth. These yield the precision and recall results for CNVnator in Supplementary Table 6.

			Ground Truth		
TOOL	Coverage	**Predicted**	NO CALL	DUP	DEL
CNVnator	Original Coverage	NO CALL	1328288	0	0
		DUP	0	12979	0
		DEL	0	0	7542
	5x	NO CALL	1326757	2253	657
		DUP	1005	10717	0
		DEL	526	9	6885
	1x	NO CALL	1326400	6927	1665
		DUP	827	6017	1
		DEL	1061	35	5876
	0.5x	NO CALL	1325884	9700	2482
		DUP	1028	3266	2
		DEL	1376	13	5058
	0.1x	NO CALL	1325883	12448	4389
		DUP	838	477	0
		DEL	1567	54	3153
	0.05x	NO CALL	1327186	12861	4813
		DUP	1	99	0
		DEL	1101	19	2729

Supplementary Table 9. Confusion Matrices for the performance comparison of CNVnator on down-sampled ancient samples (test set) in gene regions. CNVnator calls on original coverage samples are used as the ground truth. These yield the precision and recall results for CNVnator in Supplementary Table 7.

			**Ground Truth**		
TOOL	Coverage	**Predicted**	NO CALL	DUP	DEL
CNVnator	Original Coverage	NO CALL	127963	0	0
		DUP	0	1305	0
		DEL	0	0	1093
	5x	NO CALL	127786	242	161
		DUP	95	1047	0
		DEL	82	16	932
	1x	NO CALL	127751	811	353
		DUP	63	481	1
		DEL	149	13	739
	0.5x	NO CALL	127615	1014	499
		DUP	114	286	1
		DEL	234	5	593
	0.1x	NO CALL	127705	1226	768
		DUP	95	66	0
		DEL	163	13	325
	0.05x	NO CALL	127834	1284	810
		DUP	0	17	0
		DEL	129	4	283

Supplementary Table 10. Confusion Matrices for the performance comparison of CONGA on down-sampled ancient samples (test set) in exon regions. CNVnator calls on original coverage samples are used as the ground truth. These yield the precision and recall results for CONGA in Supplementary Table 6.

			**Ground Truth**		
TOOL	Coverage	**Predicted**	NO CALL	DUP	DEL
CONGA	Original Coverage	NO CALL	1328062	12852	6842
		DUP	103	115	0
		DEL	123	12	700
	5x	NO CALL	1328068	12862	6835
		DUP	92	105	0
		DEL	128	12	707
	1x	NO CALL	1327966	12852	6808
		DUP	113	115	0
		DEL	209	12	734
	0.5x	NO CALL	1327773	12862	6811
		DUP	114	105	0
		DEL	401	12	731
	0.1x	NO CALL	1325498	12886	6816
		DUP	802	78	0
		DEL	1988	15	726
	0.05x	NO CALL	1323509	12906	6896
		DUP	1429	57	0
		DEL	3350	16	646

Supplementary Table 11. Confusion Matrices for the performance comparison of CONGA on down-sampled ancient samples (test set) in gene regions. CNVnator calls on original coverage samples are used as the ground truth. These yield the precision and recall results for CONGA in Supplementary Table 7.

			Ground Truth		
TOOL	Coverage	**Predicted**	NO CALL	DUP	DEL
CONGA	Original Coverage	NO CALL	127912	1276	957
		DUP	15	19	0
		DEL	36	10	136
	5x	NO CALL	127912	1277	958
		DUP	14	18	0
		DEL	37	10	135
	1x	NO CALL	127861	1276	955
		DUP	17	19	0
		DEL	85	10	138
	0.5x	NO CALL	127763	1277	959
		DUP	22	18	0
		DEL	178	10	134
	0.1x	NO CALL	126875	1280	955
		DUP	195	15	0
		DEL	893	10	138
	0.05x	NO CALL	126242	1282	974
		DUP	324	10	0
		DEL	1397	13	119

Supplementary Table 12. Confusion Matrices for the performance comparison of Control-FREEC on down-sampled ancient samples (test set) in exon regions. CNVnator calls on original coverage samples are used as the ground truth. These yield the precision and recall results for Control-FREEC in Supplementary Table 6.

			Ground Truth		
TOOL	Coverage	**Predicted**	NO CALL	DUP	DEL
Control-FREEC	Original Coverage	NO CALL	1297701	2483	1315
		DUP	1771	10989	0
		DEL	29212	29	6261
	5x	NO CALL	1297972	2495	1293
		DUP	1351	10858	1
		DEL	29361	148	6282
	1x	NO CALL	1297338	3911	1778
		DUP	1716	9363	1
		DEL	29630	227	5797
	0.5x	NO CALL	1297449	5324	2607
		DUP	1661	8010	4
		DEL	29574	167	4965
	0.1x	NO CALL	1296735	8279	4227
		DUP	2785	4679	12
		DEL	29164	543	3337
	0.05x	NO CALL	1295987	8894	4597
		DUP	3845	4159	27
		DEL	28852	448	2952

Supplementary Table 13. Confusion Matrices for the performance comparison of Control-FREEC on down-sampled ancient samples (test set) in gene regions. CNVnator calls on original coverage samples are used as the ground truth. These yield the precision and recall results for Control-FREEC in Supplementary Table 7.

			**Ground Truth**		
TOOL	Coverage	**Predicted**	NO CALL	DUP	DEL
Control-FREEC	Original Coverage	NO CALL	124588	257	282
		DUP	155	1015	1
		DEL	3213	20	809
	5x	NO CALL	124598	279	282
		DUP	125	985	0
		DEL	3233	28	810
	1x	NO CALL	124549	432	396
		DUP	142	824	1
		DEL	3265	36	695
	0.5x	NO CALL	124566	630	515
		DUP	135	635	1
		DEL	3255	27	576
	0.1x	NO CALL	124442	846	724
		DUP	289	356	2
		DEL	3225	90	366
	0.05x	NO CALL	124356	864	784
		DUP	409	344	4
		DEL	3191	84	304

Supplementary Table 14. Confusion Matrices for the performance comparison of GATK on down-sampled ancient samples (test set) in exon regions. CNVnator calls on original coverage samples are used as the ground truth. These yield the precision and recall results for GATK in Supplementary Table 6.

			**Ground Truth**		
TOOL	Coverage	**Predicted**	NO CALL	DUP	DEL
GATK	Original Coverage	NO CALL	1293972	2672	750
		DUP	487	168	66
		DEL	33411	9739	6620
	5x	NO CALL	1309054	5979	2997
		DUP	553	62	64
		DEL	18263	6538	4375
	1x	NO CALL	1306365	6539	3320
		DUP	3234	28	0
		DEL	18271	6012	4116
	0.5x	NO CALL	1279198	6930	3454
		DUP	27275	45	1
		DEL	21397	5604	3981
	0.1x	NO CALL	1241651	9291	4277
		DUP	47275	12	12
		DEL	38944	3276	3147
	0.05x	NO CALL	1235763	10732	4398
		DUP	39057	12	5
		DEL	53050	1835	3033

Supplementary Table 15. Confusion Matrices for the performance comparison of GATK on down-sampled ancient samples (test set) in gene regions. CNVnator calls on original coverage samples are used as the ground truth. These yield the precision and recall results for GATK in Supplementary Table 7.

			**Ground Truth**		
TOOL	Coverage	**Predicted**	NO CALL	DUP	DEL
GATK	Original Coverage	NO CALL	126471	857	639
		DUP	24	4	0
		DEL	1468	444	454
	5x	NO CALL	127251	967	722
		DUP	53	3	0
		DEL	659	335	371
	1x	NO CALL	127154	1018	747
		DUP	147	0	0
		DEL	662	287	346
	0.5x	NO CALL	125912	1073	744
		DUP	1137	5	6
		DEL	914	227	343
	0.1x	NO CALL	123505	1170	835
		DUP	2655	1	4
		DEL	1803	134	254
	0.05x	NO CALL	123212	1225	835
		DUP	1975	1	5
		DEL	2776	79	253

Supplementary Table 16. Confusion Matrices for the performance comparison of LYCEUM on down-sampled ancient samples (test set) in exon regions. CNVnator calls on original coverage samples are used as the ground truth. These yield the precision and recall results for LYCEUM in Supplementary Table 6.

			**Ground Truth**		
TOOL	Coverage	**Predicted**	NO CALL	DUP	DEL
LYCEUM	Original Coverage	NO CALL	1321674	3138	1784
		DUP	1806	8620	232
		DEL	4808	1221	5526
	5x	NO CALL	1321129	2996	2155
		DUP	2324	8548	259
		DEL	4835	1435	5128
	1x	NO CALL	1319088	3361	2369
		DUP	2374	8097	260
		DEL	6826	1521	4913
	0.5x	NO CALL	1322792	3100	2772
		DUP	2583	9298	321
		DEL	2913	581	4449
	0.1x	NO CALL	1321998	3280	2796
		DUP	1941	8578	207
		DEL	4349	1121	4539
	0.05x	NO CALL	1323986	3092	3590
		DUP	2704	9620	89
		DEL	1598	267	3863

Supplementary Table 17. Confusion Matrices for the performance comparison of LYCEUM on down-sampled ancient samples (test set) in gene regions. CNVnator calls on original coverage samples are used as the ground truth. These yield the precision and recall results for LYCEUM in Supplementary Table 7.

			Ground Truth		
TOOL	Coverage	**Predicted**	NO CALL	DUP	DEL
LYCEUM	Original Coverage	NO CALL	126764	256	280
		DUP	251	875	36
		DEL	948	174	777
	5x	NO CALL	126767	264	311
		DUP	317	850	37
		DEL	879	191	745
	1x	NO CALL	126463	267	296
		DUP	509	858	41
		DEL	991	180	756
	0.5x	NO CALL	126898	280	350
		DUP	406	919	47
		DEL	659	106	696
	0.1x	NO CALL	126906	282	373
		DUP	273	856	25
		DEL	784	167	695
	0.05x	NO CALL	127269	287	474
		DUP	348	957	15
		DEL	346	61	604

Supplementary Table 18. The performance comparison of LYCEUM on 7 moderate coverage real ancient samples samples at their original coverage, categorized by their contamination levels. The c values indicate contamination predictions obtained using ContaminationX. Refer to Supplementary Table 29 for the detailed ContaminationX output for the test set.

	**DEL Precision**	**DEL Recall**	**DEL F1**	**DUP Precision**	**DUP Recall**	**DUP F1**	**Overall Precision**	**Overall Recall**	**Overall F1**
Low Contamination ($c < 1\%$)	0.398	0.788	0.529	0.825	0.664	0.736	0.611	0.726	0.632
High Contamination ($c > 30\%$)	0.594	0.686	0.637	0.788	0.665	0.721	0.691	0.675	0.679

Supplementary Table 19. The performance comparison of the CNV callers on simulated ancient samples (test set) in exon regions.

**Coverage**	**Tools**	**DEL Precision**	**DEL Recall**	**DEL F1 Score**	**DUP Precision**	**DUP Recall**	**DUP F1 Score**	**Overall Precision**	**Overall Recall**	**Overall F1 Score**
1x	CNVnator	0.854	0.545	0.665	0.834	0.145	0.248	0.844	0.345	0.457
	CONGA	0.797	0.114	0.199	0.742	0.122	0.21	0.77	0.118	0.205
	Control-FREEC	0.027	0.144	0.045	0.852	0.03	0.058	0.44	0.087	0.052
	LYCEUM	0.924	0.811	0.864	0.942	0.949	0.945	0.933	0.88	0.905
0.5x	CNVnator	0.79	0.381	0.514	0.666	0.029	0.055	0.728	0.205	0.285
	CONGA	0.747	0.111	0.194	0.738	0.12	0.206	0.743	0.116	0.2
	Control-FREEC	0.023	0.126	0.04	0.757	0.029	0.055	0.39	0.078	0.048
	LYCEUM	0.952	0.763	0.847	0.93	0.942	0.936	0.941	0.853	0.892
0.1x	CNVnator	0.387	0.052	0.091	0.201	0.018	0.034	0.294	0.035	0.063
	CONGA	0.419	0.108	0.171	0.641	0.113	0.192	0.53	0.111	0.182
	Control-FREEC	0.022	0.12	0.038	0.558	0.023	0.045	0.29	0.072	0.042
	LYCEUM	0.922	0.778	0.844	0.934	0.929	0.932	0.928	0.854	0.888
0.05x	CNVnator	0.616	0.025	0.049	0.175	0.004	0.007	0.396	0.015	0.028
	CONGA	0.275	0.102	0.149	0.556	0.109	0.182	0.416	0.106	0.166
	Control-FREEC	0.017	0.091	0.029	0.432	0.017	0.032	0.225	0.054	0.031
	LYCEUM	0.935	0.721	0.814	0.92	0.901	0.91	0.928	0.811	0.862

Supplementary Table 20. The performance comparison of the CNV callers on simulated ancient samples (test set) in gene regions.

Coverage	Tools	DEL Precision	DEL Recall	DEL F1 Score	DUP Precision	DUP Recall	DUP F1 Score	Overall Precision	Overall Recall	Overall F1 Score
1x	CNVnator	0.839	0.438	0.576	0.916	0.096	0.173	0.878	0.267	0.375
	CONGA	0.753	0.174	0.283	0.86	0.142	0.244	0.807	0.158	0.264
	Control-FREEC	0.5	0.0	0.001	0.833	0.006	0.012	0.667	0.003	0.007
	LYCEUM	0.922	0.758	0.832	0.952	0.93	0.941	0.937	0.844	0.887
0.5x	CNVnator	0.736	0.269	0.394	0.861	0.027	0.051	0.799	0.148	0.223
	CONGA	0.678	0.173	0.275	0.858	0.14	0.241	0.768	0.157	0.258
	Control-FREEC	0.5	0.0	0.001	0.442	0.007	0.014	0.471	0.004	0.008
	LYCEUM	0.952	0.712	0.815	0.927	0.921	0.924	0.94	0.817	0.87
0.1x	CNVnator	0.207	0.01	0.02	0.48	0.024	0.046	0.344	0.017	0.033
	CONGA	0.315	0.17	0.221	0.711	0.133	0.225	0.513	0.152	0.223
	Control-FREEC	0.0	0.0	0.0	0.339	0.004	0.008	0.17	0.002	0.004
	LYCEUM	0.917	0.714	0.803	0.931	0.898	0.914	0.924	0.806	0.859
0.05x	CNVnator	0.0	0.0	0.0	0.472	0.005	0.01	0.236	0.003	0.005
	CONGA	0.215	0.163	0.185	0.579	0.126	0.207	0.397	0.145	0.196
	Control-FREEC	0.0	0.0	0.0	0.571	0.003	0.006	0.286	0.002	0.003
	LYCEUM	0.923	0.682	0.785	0.905	0.869	0.886	0.914	0.776	0.836

Supplementary Table 21. Confusion Matrices for the performance comparison of CNVnator on simulated ancient samples (test set) in exon regions. These yield the precision and recall results for CNVnator in Supplementary Table 19.

			**Ground Truth**		
TOOL	Coverage	**Predicted**	NO CALL	DUP	DEL
CNVnator	1x	NO CALL	1881605	24403	6554
		DUP	825	4150	0
		DEL	1335	0	7838
	0.5x	NO CALL	1881976	27652	8907
		DUP	409	821	2
		DEL	1380	80	5483
	0.1x	NO CALL	1880539	28021	13630
		DUP	2057	522	18
		DEL	1169	10	744
	0.05x	NO CALL	1883037	28446	14024
		DUP	501	107	4
		DEL	227	0	364

Supplementary Table 22. Confusion Matrices for the performance comparison of CNVnator on simulated ancient samples (test set) in gene regions. These yield the precision and recall results for CNVnator in Supplementary Table 20.

			**Ground Truth**		
TOOL	Coverage	**Predicted**	NO CALL	DUP	DEL
CNVnator	1x	NO CALL	178083	4410	1670
		DUP	41	469	2
		DEL	232	19	1304
	0.5x	NO CALL	178066	4749	2175
		DUP	21	130	0
		DEL	269	19	801
	0.1x	NO CALL	178114	4775	2945
		DUP	128	118	0
		DEL	114	5	31
	0.05x	NO CALL	178318	4873	2976
		DUP	28	25	0
		DEL	10	0	0

Supplementary Table 23. Confusion Matrices for the performance comparison of CONGA on simulated ancient samples (test set) in exon regions. These yield the precision and recall results for CONGA in Supplementary Table 19.

			**Ground Truth**		
TOOL	Coverage	**Predicted**	NO CALL	DUP	DEL
CONGA	1x	NO CALL	1882136	25063	12752
		DUP	1212	3490	0
		DEL	417	0	1640
	0.5x	NO CALL	1882012	25129	12787
		DUP	1211	3424	2
		DEL	542	0	1603
	0.1x	NO CALL	1879816	25331	12842
		DUP	1802	3221	2
		DEL	2147	1	1548
	0.05x	NO CALL	1877427	25438	12919
		DUP	2480	3106	4
		DEL	3858	9	1469

Supplementary Table 24. Confusion Matrices for the performance comparison of CONGA on simulated ancient samples (test set) in gene regions. These yield the precision and recall results for CONGA in Supplementary Table 20.

			**Ground Truth**		
TOOL	Coverage	**Predicted**	NO CALL	DUP	DEL
CONGA	1x	NO CALL	178121	4184	2426
		DUP	82	697	31
		DEL	153	17	519
	0.5x	NO CALL	178045	4197	2432
		DUP	83	685	30
		DEL	228	16	514
	0.1x	NO CALL	177047	4210	2445
		DUP	242	653	24
		DEL	1067	35	507
	0.05x	NO CALL	176194	4243	2466
		DUP	425	618	25
		DEL	1737	37	485

Supplementary Table 25. Confusion Matrices for the performance comparison of Control-FREEC on simulated ancient samples (test set) in exon regions. These yield the precision and recall results for Control-FREEC in Supplementary Table 19.

			**Ground Truth**		
TOOL	Coverage	**Predicted**	NO CALL	DUP	DEL
Control-FREEC	1x	NO CALL	1809002	28061	12331
		DUP	147	868	4
		DEL	75386	0	2081
	0.5x	NO CALL	1808909	28101	12604
		DUP	264	826	1
		DEL	75362	2	1811
	0.1x	NO CALL	1808363	28246	12693
		DUP	537	678	0
		DEL	75635	5	1723
	0.05x	NO CALL	1808029	28445	13099
		DUP	635	482	0
		DEL	75871	2	1317

Supplementary Table 26. Confusion Matrices for the performance comparison of Control-FREEC on simulated ancient samples (test set) in gene regions. These yield the precision and recall results for Control-FREEC in Supplementary Table 20.

			Ground Truth		
TOOL	Coverage	**Predicted**	NO CALL	DUP	DEL
Control-FREEC	1x	NO CALL	178319	4868	2975
		DUP	6	30	0
		DEL	1	0	1
	0.5x	NO CALL	178282	4864	2975
		DUP	43	34	0
		DEL	1	0	1
	0.1x	NO CALL	178289	4879	2976
		DUP	37	19	0
		DEL	0	0	0
	0.05x	NO CALL	178314	4882	2976
		DUP	12	16	0
		DEL	0	0	0

Supplementary Table 27. Confusion Matrices for the performance comparison of LYCEUM on simulated ancient samples (test set) in exon regions. These yield the precision and recall results for LYCEUM in Supplementary Table 19.

			Ground Truth		
TOOL	Coverage	**Predicted**	NO CALL	DUP	DEL
LYCEUM	1x	NO CALL	1881256	1422	2625
		DUP	1580	27096	88
		DEL	929	35	11679
	0.5x	NO CALL	1881315	1659	3285
		DUP	1902	26883	120
		DEL	548	11	10987
	0.1x	NO CALL	1881113	1970	3106
		DUP	1767	26521	94
		DEL	885	62	11192
	0.05x	NO CALL	1880964	2763	3912
		DUP	2144	25728	103
		DEL	657	62	10377

Supplementary Table 28. Confusion Matrices for the performance comparison of LYCEUM on simulated ancient samples (test set) in gene regions. These yield the precision and recall results for LYCEUM in Supplementary Table 20.

			Ground Truth		
TOOL	Coverage	**Predicted**	NO CALL	DUP	DEL
LYCEUM	1x	NO CALL	177974	328	694
		DUP	205	4556	26
		DEL	177	14	2256
	0.5x	NO CALL	177950	380	810
		DUP	307	4510	47
		DEL	99	8	2119
	0.1x	NO CALL	177888	476	823
		DUP	298	4400	28
		DEL	170	22	2125
	0.05x	NO CALL	177797	627	904
		DUP	407	4254	41
		DEL	152	17	2031

Supplementary Table 29. ContaminationX output for 7 moderate coverage real ancient samples at their original coverage levels.

Sample Name	Method	ContaminationEstimate	LowerCI	UpperCI	ErrorRate	SitesUsed
Nea3	One-cns	0.3924	0.3919	0.3930	0.0011	55756
	Two-cns	0.3974	0.3969	0.3979	0.0011	55756
AKT16	One-cns	0.3697	0.3691	0.3703	0.0012	52695
	Two-cns	0.3779	0.3773	0.3784	0.0012	52695
STAR1	One-cns	0.3665	0.3660	0.3670	0.0011	56199
	Two-cns	0.3736	0.3731	0.3741	0.0011	56199
VLASA7	One-cns	0.0057	0.0056	0.0058	0.0009	55396
	Two-cns	0.0058	0.0057	0.0059	0.0009	55396
Bichon	One-cns	0.0031	0.0029	0.0032	0.0029	38725
	Two-cns	0.0032	0.0030	0.0033	0.0029	38725
VLASA32	One-cns	0.0064	0.0063	0.0065	0.0012	51731
	Two-cns	0.0064	0.0064	0.0065	0.0012	51731
Ust	One-cns	0.0040	0.0040	0.0041	0.0013	37174
	Two-cns	0.0040	0.0040	0.0041	0.0013	37174

Supplementary Table 30. Performance comparison of the LYCEUM architecture with and without Transformer blocks on original coverage real samples and 5× simulated data.

		DEL F1 Score	DUP F1 Score	Overall F1 Score
Without Transformer	Real Samples	0.344	0.208	0.276
	Simulated Samples	0.017	0.054	0.035
With Transformer	Real Samples	0.579	0.729	0.654
	Simulated Samples	0.864	0.945	0.904

3 Supplemantary Notes

3.1 Detailed Model Architecture

Input Representation: For each exon X^i, the model inputs a normalized read depth signal, $X^i_{RD} \in \mathbb{R}^{1000\times 1}$. Each input vector represents a fixed-length (up to 1 kbp) segment. We left-pad shorter signals, and discard exons exceeding 1 kbp to maintain input sizes uniformly.

Convolutional Feature Extraction: The initial step of LYCEUM uses two sequential 1D convolutional layers to extract local features from the input signal:

- **Convolution Layer 1:**
  - **Input Channels:** 1
  - **Output Channels:** 32
  - **Kernel Size:** 3, **Stride:** 1
  - **Activation:** Batch Normalization (BN) followed by ReLU
  - **Output:** A feature map of dimensions $\mathbb{R}^{1000\times 32}$.
- **Convolution Layer 2:**
  - **Input Channels:** 32
  - **Output Channels:** 64
  - **Kernel Size:** 3, **Stride:** 1
  - **Activation:** Batch Normalization (BN) and ReLU
  - **Output:** The resulting feature map is $X^i_{RDE} \in \mathbb{R}^{1000\times 64}$.

Classification Token: We define a trainable classification token $c^t \in \mathbb{R}^{64}$ for each chromosome t. We add this token to the convolution output along the sequence length dimension, which results in the following tensor:

$$\hat{\mathbf{X}}^i_{RDE} \in \mathbb{R}^{64\times 1001},$$

where the additional column (position 1001) represents the chromosome-specific token. This token incorporates context from the exon and we used it later for classification.

Positional Encoding: To maintain positional information, we add a learnable positional encoding tensor of the same shape as $\hat{\mathbf{X}}^i_{RDE}$ element-wise. This encoding ensures that the model preserves the spatial ordering of the read depth values throughout.

By providing the model with positional information of exons along with the read depth, it learns position-specific read depth characteristics, including intrinsic biases arising from GC content or repetitive sequences. As a result, the model becomes aware of deviations in exon read depths when they are frequently observed during training, eliminating the need for separate correction steps like GC-correction (see Supplementary Figures 1-2).

Transformer Encoder Blocks: We then pass the augmented tensor through a cascade of three transformer encoder blocks. Each block includes:

- **Multi-Head Self-Attention:** Captures long-range dependencies across the exon. We use 8 heads.
- **Layer Normalization and Residual Connections:** Applied around both the attention and feed-forward components to ensure stable training.
- **Position-wise Feed-Forward Network (FFN):** Applied at each position independently, with a hidden dimension typically 2–4 times the embedding size.

The output tensor from the final transformer block is $\mathbf{O}^i_{RDE} \in \mathbb{R}^{64\times 1001}$.

Classification Head: We extract the enriched classification token from the final output:

$$\mathbf{z}^i = \mathbf{O}^i_{RDE}(:, 1001) \in \mathbb{R}^{64}.$$

We pass this vector through a fully connected layer that maps it to a 3-dimensional output corresponding to the classes: deletion (DEL), duplication (DUP), and no-call (NO-CALL). We apply a softmax function to produce class probabilities:

$$\hat{Y}^i = \text{softmax}(\mathbf{W}\mathbf{z}^i + \mathbf{b}),$$

where $\mathbf{W}$ and $\mathbf{b}$ are learnable parameters.

Majority Voting for Missing Data: Approximately 5% of exonic regions in aDNA samples lack sufficient read-depth information. For these cases, LYCEUM employs a majority voting mechanism:

– **Exon-Level:** We obtain the CNV prediction by taking the majority vote among the three nearest-neighbor exons.
– **Gene-Level:** For gene-level calls, we perform a majority vote across all exons within a gene to ensure robust predictions despite missing data.

3.2 Data Format and Preprocessing

Input Data Format LYCEUM accepts as input a normalized read depth signal extracted from whole-genome sequencing (WGS) or ancient DNA (aDNA) datasets. For each exon X^i, the input consists of:

– A normalized read depth vector $X^i_{RD} \in \mathbb{R}^{1000\times 1}$ representing the sequencing coverage over the exon region.
– Associated metadata, including the chromosome identifier X^i_{ch} and genomic coordinates X^i_{start} and X^i_{end}.

The fixed-length vector (1000 elements) is created by extracting the read depth over the exon, left-padding exons shorter than 1 kbp, and discarding exons longer than 1 kbp.

Preprocessing Pipeline Our pipeline accepts raw alignment files in `.bam` format and preprocesses them using the following steps. Once the data has been processed, it is ready for CNV calling with LYCEUM.

1. **BAM File Indexing and Read Depth Generation:**
Raw BAM files are first indexed using `samtools index`. Next, the read depth is computed over target regions specified in a BED file (e.g., `hg38_hglft_genome_64dc_dcbaa0_unique.bed`) using the `sambamba depth base` command. This generates text files containing base-level read depth information for each exon, which are stored in a designated directory (e.g., `read_depths/`).
2. **Exon-Level Read Depth Extraction**
It reads the base-level read depth data and the target exon BED file. For each sample, the script iterates over the target exons and extracts the corresponding read depth values. The extracted read depth signal is assembled into a vector whose length matches the exon size. Exons are left-padded if shorter than 1 kbp, and exons longer than 1 kbp are discarded. Each processed exon, along with its metadata (sample name, chromosome, start, and end coordinates), is stored as a labeled NumPy array file.
3. **Calculation of Statistics**
Finally, it computes summary statistics for the processed samples. It concatenates all read depth vectors from a given sample and calculates the mean and standard deviation. A lookup table is generated (stored as a NumPy file) that maps each sample to its corresponding mean and standard deviation. This lookup table is used for further normalization or quality control.

3.3 Ablation Study

LYCEUM is built upon a transformer-based architecture, featuring three transformer blocks at its core. These blocks, combined with positional encoding, allow the model to effectively capture relationships between read depth values across all base pairs within a given exon sample. To evaluate the impact of the transformer blocks, we ablated them from the architecture and performed both pretraining and finetuning using the same dataset as used for LYCEUM. Our results show that incorporating transformer blocks leads to substantial improvements in overall F1 score—38% for real samples and 87% for simulated samples (see Supplementary Table 30).

Supplementary References

1. Morten Rasmussen, Sarah L Anzick, Michael R Waters, Pontus Skoglund, Michael DeGiorgio, Thomas W Stafford Jr, Simon Rasmussen, Ida Moltke, Anders Albrechtsen, Shane M Doyle, et al. The genome of a late pleistocene human from a clovis burial site in western montana. *Nature*, 506(7487):225–229, 2014.
2. Nina Marchi, Laura Winkelbach, Ilektra Schulz, Maxime Brami, Zuzana Hofmanová, Jens Blöcher, Carlos S Reyna-Blanco, Yoan Diekmann, Alexandre Thiéry, Adamandia Kapopoulou, et al. The mixed genetic origin of the first farmers of europe. *bioRxiv*, pages 2020–11, 2020.
3. Peter de Barros Damgaard, Nina Marchi, Simon Rasmussen, Michaël Peyrot, Gabriel Renaud, Thorfinn Korneliussen, J Víctor Moreno-Mayar, Mikkel Winther Pedersen, Amy Goldberg, Emma Usmanova, et al. 137 ancient human genomes from across the eurasian steppes. *Nature*, 557(7705):369–374, 2018.
4. Gülşah Merve Kılınç, Natalija Kashuba, Dilek Koptekin, Nora Bergfeldt, Handan Melike Dönertaş, Ricardo Rodríguez-Varela, Dmitrij Shergin, Grigorij Ivanov, Dmitrii Kichigin, Kjunnej Pestereva, et al. Human population dynamics and yersinia pestis in ancient northeast asia. *Science Advances*, 7(2):eabc4587, 2021.
5. Eppie R Jones, Gloria Gonzalez-Fortes, Sarah Connell, Veronika Siska, Anders Eriksson, Rui Martiniano, Russell L McLaughlin, Marcos Gallego Llorente, Lara M Cassidy, Cristina Gamba, et al. Upper palaeolithic genomes reveal deep roots of modern eurasians. *Nature communications*, 6(1):8912, 2015.
6. Iosif Lazaridis, Nick Patterson, Alissa Mittnik, Gabriel Renaud, Swapan Mallick, Karola Kirsanow, Peter H Sudmant, Joshua G Schraiber, Sergi Castellano, Mark Lipson, et al. Ancient human genomes suggest three ancestral populations for present-day europeans. *Nature*, 513(7518):409–413, 2014.
7. Farnaz Broushaki, Mark G Thomas, Vivian Link, Saioa López, Lucy Van Dorp, Karola Kirsanow, Zuzana Hofmanová, Yoan Diekmann, Lara M Cassidy, David Díez-del Molino, et al. Early neolithic genomes from the eastern fertile crescent. *Science*, 353(6298):499–503, 2016.
8. Qiaomei Fu, Heng Li, Priya Moorjani, Flora Jay, Sergey M Slepchenko, Aleksei A Bondarev, Philip LF Johnson, Ayinuer Aximu-Petri, Kay Prüfer, Cesare De Filippo, et al. Genome sequence of a 45,000-year-old modern human from western siberia. *Nature*, 514(7523):445–449, 2014.
9. Gülşah Merve Kılınç, Ayça Omrak, Füsun Özer, Torsten Günther, Ali Metin Büyükkarakaya, Erhan Bıçakçı, Douglas Baird, Handan Melike Dönertaş, Ayshin Ghalichi, Reyhan Yaka, et al. The demographic development of the first farmers in anatolia. *Current Biology*, 26(19):2659–2666, 2016.
10. Margaret L Antonio, Ziyue Gao, Hannah M Moots, Michaela Lucci, Francesca Candilio, Susanna Sawyer, Victoria Oberreiter, Diego Calderon, Katharina Devitofranceschi, Rachael C Aikens, et al. Ancient rome: A genetic crossroads of europe and the mediterranean. *Science*, 366(6466):708–714, 2019.
11. Torsten Günther, Cristina Valdiosera, Helena Malmström, Irene Ureña, Ricardo Rodriguez-Varela, Óddny Osk Sverrisdóttir, Evangelia A Daskalaki, Pontus Skoglund, Thijessen Naidoo, Emma M Svensson, et al. Ancient genomes link early farmers from atapuerca in spain to modern-day basques. *Proceedings of the National Academy of Sciences*, 112(38):11917–11922, 2015.
12. Z Hofmanová, S Kreutzer, G Hellenthal, C Sell, Y Diekmann, D Díez-Del-Molino, and J Burger. Early farmers from across europe directly descended from neolithic aegeans. pnas, 2016.
13. Martin Sikora, Vladimir V Pitulko, Vitor C Sousa, Morten E Allentoft, Lasse Vinner, Simon Rasmussen, Ashot Margaryan, Peter de Barros Damgaard, Constanza de la Fuente, Gabriel Renaud, et al. The population history of northeastern siberia since the pleistocene. *Nature*, 570(7760):182–188, 2019.
14. Morten E Allentoft, Martin Sikora, Karl-Göran Sjögren, Simon Rasmussen, Morten Rasmussen, Jesper Stenderup, Peter B Damgaard, Hannes Schroeder, Torbjörn Ahlström, Lasse Vinner, et al. Population genomics of bronze age eurasia. *Nature*, 522(7555):167–172, 2015.
15. Maja Krzewińska, Gülşah Merve Kılınç, Anna Juras, Dilek Koptekin, Maciej Chyleński, Alexey G Nikitin, Nikolai Shcherbakov, Iia Shuteleva, Tatiana Leonova, Liudmila Kraeva, et al. Ancient genomes suggest the eastern pontic-caspian steppe as the source of western iron age nomads. *Science advances*, 4(10):eaat4457, 2018.
16. Peter de Barros Damgaard, Rui Martiniano, Jack Kamm, J Víctor Moreno-Mayar, Guus Kroonen, Michaël Peyrot, Gojko Barjamovic, Simon Rasmussen, Claus Zacho, Nurbol Baimukhanov, et al. The first horse herders and the impact of early bronze age steppe expansions into asia. *Science*, 360(6396):eaar7711, 2018.
17. Gloria González-Fortes, Eppie R Jones, Emma Lightfoot, Clive Bonsall, Catalin Lazar, Aurora Grandal-d'Anglade, María Dolores Garralda, Labib Drak, Veronika Siska, Angela Simalcsik, et al. Paleogenomic evidence for multi-generational mixing between neolithic farmers and mesolithic hunter-gatherers in the lower danube basin. *Current Biology*, 27(12):1801–1810, 2017.
18. Reyhan Yaka, Igor Mapelli, Damla Kaptan, Ayça Doğu, Maciej Chyleński, Ömür Dilek Erdal, Dilek Koptekin, Kıvılcım Başak Vural, Alex Bayliss, Camilla Mazzucato, et al. Variable kinship patterns in neolithic anatolia revealed by ancient genomes. *Current Biology*, 31(11):2455–2468, 2021.
19. Rui Martiniano, Lara M Cassidy, Ros Ó'Maoldúin, Russell McLaughlin, Nuno M Silva, Licinio Manco, Daniel Fidalgo, Tania Pereira, Maria J Coelho, Miguel Serra, et al. The population genomics of archaeological transition in west iberia: Investigation of ancient substructure using imputation and haplotype-based methods. *PLoS genetics*, 13(7):e1006852, 2017.
